# Understanding gender differences in professional European football through machine learning interpretability and match actions data

**DOI:** 10.1038/s41598-021-90264-w

**Published:** 2021-05-24

**Authors:** Marc Garnica-Caparrós, Daniel Memmert

**Affiliations:** Institute of Training and Computer Science in Sport, German Sport University Cologne, Am Sportpark Müngersdorf 6, 50933 Cologne, Germany

**Keywords:** Computer science, Information technology, Scientific data, Software, Statistics

## Abstract

After the great success of the Women’s World Cup in 2019, several platforms have started identifying the reasons for gender inequality in European football. Even though these inequalities emerge from a variety of key aspects in the modern sport, we focused on the game and evaluated the main differential features of European male and female football players in match actions data under the assumption of finding significant differences and established patterns between genders. A methodology for unbiased feature extraction and objective analysis is presented based on data integration and machine learning explainability algorithms. Female ($n_0 = 1511$) and male ($n_1 = 2703$) data points were collected from event data and categorized by game period and player position. We set up a supervised classification pipeline to predict the gender of each player by looking at their actions in the game. The comparison methodology did not include any qualitative enrichment or subjective analysis to prevent biased data enhancement or gender-related processing. The pipeline included three representative binary classification models; A logic-based Decision Trees, a probabilistic Logistic Regression and a multilevel perceptron Neural Network. Each model tried to draw the differences between male and female data points, and we extracted the results using machine learning explainability methods to understand the underlying mechanics of the models implemented. The study was able to determine pivotal factors that differentiate each gender performance as well as disseminate unique patterns by gender involving more than one indicator. Data enhancement and critical variables analysis are essential next steps to support this framework and serve as a baseline for further studies and training developments.

## Introduction

Data modelling and analysis have become a powerful and differential tool in modern European football. Many professional teams currently rely on the use of data, technology and statistics to support their operations. The optimised results and conclusions from these innovative methods help professional clubs in scouting, management and performance. Professional players are now evaluated and compared using a comprehensive list of performance indicators to determine their value and contribution to the team^1–3^. These indicators have also shown the ability to determine the style of play of specific teams^4^ and numerous applications for sports forecasting^5^. Despite the undoubtedly increasing data-based culture in football, there are still certain factors in this sport that are based on subjective and biased criteria. Unfortunately, there is still a prevalent fan and media-based opinion that male performances are systematically better than female performances in a collaborative sport such as football and hence, more attractive. After the great success in FIFA Women’s World Cup in 2019, gender inequality in football has been a highly addressed and traversal topic. Even though these inequalities emerge from various key aspects in modern sports, in the present study, we aim to evaluate the game and apply state-of-the-art sports analysis methods to determine the differences between male and female European football.

The scientific community has led the proliferation of football analytics metrics and methods during the last decades. Tracking technologies based on sensor devices and video analysis tools stream high-frequency data covering all aspects of the game^6^. The amount of data every football game generates at the team and player level is growing exponentially. The detailed collection of every single action occurring in a match is often called event data or also referred to in the European football community as soccer-logs^7^. Event data allows for an extensive and flexible description of team and players performance and sequential pattern analysis^8^. Beyond mathematical and statistical models, machine learning models have also been presented in recent years leveraging football event data^9,10^.

Studying differences between male and female athletes in sports environments is a primarily addressed topic in sports medicine under different perspectives such as participation, motivation, or biomechanics studies. In European football, broadcast recording analysis reported significant differences between player movement patterns by gender^11^. Other studies showed similar results under the limitation of observation protocols^12^. Video analysis and collection of ball actions data identified female’s performance as less aggressive, with less contact and longer passes. Other studies investigated female’s physical performances compared to male players from two perspectives: physical load and playing distance to the ball. Female players were found to report smaller high speed thresholds and distance coverage than male players^13^. Similarly, female movement patterns showed less prune to create spaces, hence, good penetrations into the opponent’s area^14^. A recent study confirmed previous findings analyzing the first division in Spain post match statistics^15^. Female players performed less passes with less accuracy and female performances were less controlled with a higher number of divided balls.

While previous literature presented physiological and technical initial findings differentiating female and male performances, we aim to contribute to this research by providing an analysis at the finest granularity possible, match events, and complement the comparative numerical analysis with intervariable relationships and pattern discovery. Profiling female and male performances using more than one variable for comparison could identify player styles and situations unique by gender.It is expected that using machine learning models to understand the main difference between male and female players’ performance would provide a layer of objectiveness and deeper analysis of the numerical features. In the present study, we propose a comparison framework where only raw numerical data from the data sources is used, and no other derived metrics or qualitative enhancement are included to avoid subjectivity or gender-biased data processing. Although we expect differences between male and female performances in the frequency of actions and dynamism, this study aims to understand the differences from a playing style perspective and identify unique characteristics developed specifically in each gender practice.

Machine learning techniques have demonstrated the capacity to discover underlying patterns in high dimensional data. These novel computational techniques are becoming the alternative to statistical methods computing quantitative measurements of confidence and correlation. Classification or supervised learning algorithms are an efficient tool nowadays to study and disseminate data populations into groups. Supervised learning algorithms rely on already known labels or classes to be able to predict unlabeled data. The resulting classifiers concisely draw boundaries and induce logic from the feature space to disseminate the data into labels. Explainability and interpretability are two factors of machine learning that drive the focus not only on the results but also on the data’s inherent patterns and the ability of the algorithms to explain them. To achieve the presented goal of this study, we explore how novel machine learning techniques can provide unbiased results identifying gender difference and extract underlying knowledge from the algorithms behaviours. The presented methodology uses multiple supervised classification algorithms to predict the player’s gender from the game’s actions. We evaluated each model to ensure a fitted classification, and we made use of explainability methods^16^ to induce significant differences identified by the models. In detail, we trained three representative binary classification models; A logic-based algorithm Decision Trees^17^, a probabilistic Logistic Regression model^18^, and a multi-level perceptron Neural Network^19^.

We considered for this study the two biggest stages of national competition in football in Europe. Event data sources from the 2016 UEFA Men’s European Football Championship and the 2017 UEFA Women’s Championship were obtained and processed. Key metrics by player position and gender were extracted from match event data, integrated and validated as a single dataset. We investigated the differences of female and male performances in European football to provide a purely data-driven comparison on top of any subjective dissemination. The outcome of this study should be used to enhance training programs as well as accelerate the contribution of the football analysis community in female European football. Moreover, the present methodology would enable further research on disseminating different playing styles between genders, ages, and countries of provenance and boost custom training plans and tactical supervision for male and female athletes.

## Methods

All research was performed under the relevant guidelines and regulations, and the study protocol and methodology was approved by the German Sport University of Cologne. The 2016 UEFA Men’s European Football Championship and the 2017 UEFA Women’s Championship match event data indicators were obtained through external sources. The 2016 UEFA Men’s European Football Championship is publicly available for research and analysis purposes^7^ and has been used in other contributions to present new data-driven performance metrics^10^. The 2017 UEFA Women’s Championship was provided by Opta Sports^20^ and shared by the Leuphana University of Lünenburg. Labelling each player by gender did not rely upon each player individual information but by data source provenance; all data points from the 2016 Men’s Championship were classified as male, and data points from the 2017 Women’s Championship were classified as female. In doing so, the attribution of male and female is done exclusively through the affiliation to the respective teams. No personal data was considered for this study.

**Figure 1:**
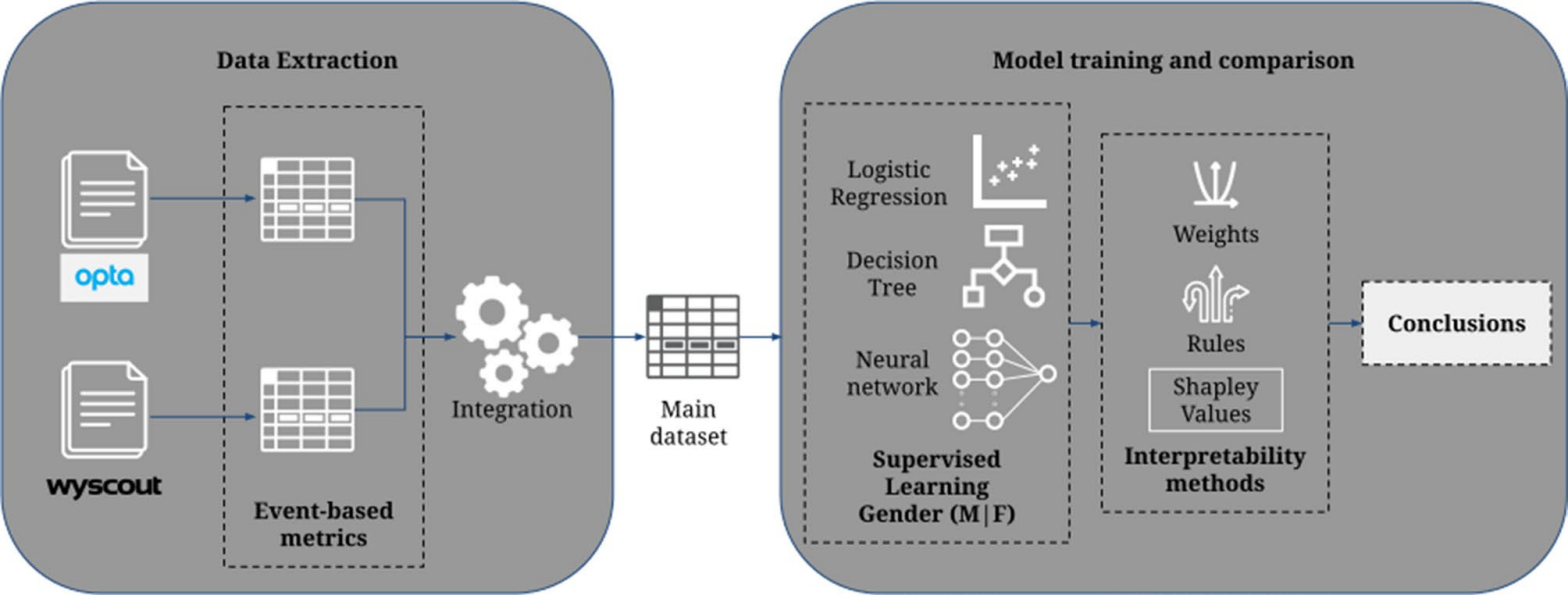
Complete methodology. We obtained event-based metrics from the two different data sources and integrated them into a single dataset. We implemented three supervised learning models and analyzed interpretability methods to understand the models.

The methodology of this study is presented in Fig. 1. We first extracted the technical indicators from the event data logs provided by the two data sources. We integrated each derived dataset into a single semantic domain and homogenised technical events names and players’ positions to build the preliminary gender labeled dataset. The integrated dataset was validated and fitted into the supervised learning models for training to identify the gender of each data point. Then, as a final step, we interpreted each classifier to understand how the gender classes disseminate data points and how the models identify these patterns. Interpretation techniques and results format varied depending on the classifier.

### Data sources

Table 1 summarizes the technical aspects of the data sources. Each data source represents all the events occurring during every match of the competition. Both sources were integrated into a schema consisting of the two competitions, and each event was defined by match and event identifier, event type and sub-type if applicable, timestamp, the player involved in the event, and the success of the event specified by accurate or not. The inference of each data source data model was performed using Apache Spark^21^ functionalities.

**Table 1:** Data sources’ specifications. Gender of the games included, data provider, the data format of the files, number of games, and number of events included.

Data source	Gender	Format	#Games	#Events
2016 UEFA Men’s Europe Championship	Male	JSON	51	74,751
2017 UEFA Women’s Europe Championship	Female	XML	31	47,976

### Feature extraction and validation

Event data collection involves a complex process of computer machinery and human annotation^6,7^. The resulting sequence of actions contains a detailed play-by-play description of the football game. Generally, each event includes its category, moment in the game, spatial information on where it occurred, if possible, and the players involved. 122727 events from the 82 matches of the two competitions were processed to compute a final list of performance indicators considering previous research on technical indicators related to match performance^22–24^. The final feature space consists of count-based metrics extracted directly from the match actions and the contextual features categorising each data point by game, game section, player, and playing position. The playing position was directly extracted from the data sources.

Event data at this granularity is often scarce in sports analytics research. In addition, the availability of these two data sets at the same edition and competition level in European football provides the study with a robust database for comparison. However, the integration of two different data providers presented challenging issues to homogenise concepts and data validation. As explained in the previous section, male players’ performance indicators were extracted from Wyscout data collection files, while female performance was extracted from Opta Sports. Several validation steps were included to avoid a systematic difference of male and female indicators by the data provider.

The two data providers presented different data formats and, more importantly, different data representation models. Female matches by Opta Sports were presented in different files per match with an additional file with the players’ information, including the playing position in the field. Each file contained a *game* object with a list of all the *events*. While the event type, time, player involved, and outcome were direct attributes of each event, the data syntaxis relied on the concept of *event qualifiers* to express the majority of event subtypes such as crosses, long passes, pass direction data, free kicks, corners, saves, headed clearances, shot categories, goals categories or cards. In contrast, male matches by Wyscout were presented in two single files containing all matches and players information, respectively. The full competition list of events contained the match, period, event type and player of every event. In this case, the accurate or inaccurate outcome of the events and certain categorisation such as long or diagonal passes were represented by *event tags*.

To ensure the definitions and concepts used in both datasets were homogeneous, we validated the coherence of each final variable within both data providers glossaries^25,26^. The selected indicators have a clear and standard definition from both data providers. However, several event categories and types were discarded due to a lack of coherence in the descriptions. Categorising match action by open and set play, pressing situations, and tackles were discarded due to the high level of human criterion they require. Similarly, possession turning events such as turnovers and ball recoveries were also eliminated because of the divergence in definitions and overlapping with other specific attributes such as over-runs or missed touches. In addition, three games from the Wyscout data source were manually validated through the publicly available data in WhoScored^27^, an online reference portal for football metrics with Opta Sports as the main data provider.

### Descriptive statistics

The resulting dataset of length $n = 4214$ contains 33 variables. The gender attribute is expressed as 1 for male players and 0 for female players. There are 2703 male and 1511 female instances. Match period is expressed as 1H for the first half, 2H for the second half, and E1, E2, and P for the two possible overtimes and the penalties respectively. Player position in the team formation has the following options: Defender, Midfielder, Forward, Goalkeeper, Substitute Defender, Substitute Midfielder, Substitute Forward and Substitute Goalkeeper. The remaining 30 variables consist of count-based statistics of each player performance during a game section. Table 2 shows the mean value and standard deviation per gender of each of the 30 numerical features of the dataset. As an initial exploratory step, male observations show a higher number of events with higher variance, denoting a higher disparity in male players styles and levels. Male players perform more and better passing in short and long distances, and they participate in more duels. On the other side, female players’ observations show higher values for fouls.

**Table 2:** Mean value and standard deviation of each variable in the dataset per gender.

Variable	Female	Male
Passes	$13.7 \pm 9.7$	$16.2 \pm 11.2$
Successful passes	$9.9 \pm 8.5$	$13.7 \pm 10.4$
Unsuccessful passes	$3.9 \pm 2.7$	$2.5 \pm 2.0$
Crosses	$0.4 \pm 0.8$	$0.7 \pm 1.1$
Successful crosses	$0.1 \pm 0.3$	$0.2 \pm 0.5$
Unsuccessful crosses	$0.4 \pm 0.7$	$0.5 \pm 0.9$
Long passes	$2.2 \pm 2.4$	$1.1 \pm 1.5$
Long passes won	$0.9 \pm 1.3$	$0.6 \pm 1.1$
Long passes lost	$1.3 \pm 1.6$	$0.5 \pm 0.8$
Aerial	$1.2 \pm 1.6$	$1.7 \pm 2.1$
Aerial won	$0.6 \pm 1.0$	$1.0 \pm 1.4$
Aerial lost	$0.6 \pm 1.0$	$0.8 \pm 1.1$
Ground duels	$2.5 \pm 2.3$	$6.1 \pm 4.3$
Ground duels won	$1.3 \pm 1.4$	$2.3 \pm 2.2$
Ground duels lost	$1.2 \pm 1.3$	$2.2 \pm 2.0$
Free kicks	$0.5 \pm 0.9$	$1.8 \pm 2.5$
Fouls received	$0.9 \pm 1.1$	$0.5 \pm 0.8$
Corners	$0.2 \pm 0.7$	$0.2 \pm 0.7$
Corners successful	$0.1 \pm 0.4$	$0.1 \pm 0.5$
Corners unsuccessful	$0.1 \pm 0.5$	$0.1 \pm 0.3$
Saves	$0.2 \pm 0.6$	$0.1 \pm 0.6$
Clearances	$1.2 \pm 1.7$	$0.6 \pm 1.1$
Interceptions	$0.5 \pm 0.9$	$1.7 \pm 1.8$
Shots	$0.5 \pm 0.8$	$0.4 \pm 0.8$
Shots on target	$0.2 \pm 0.4$	$0.1 \pm 0.4$
Shots off target	$0.2 \pm 0.5$	$0.3 \pm 0.6$
Goals	$0.0 \pm 0.2$	$0.0 \pm 0.2$
Goals from penalty	$0.0 \pm 0.1$	$0.0 \pm 0.1$
Yellow card	$0.1 \pm 0.2$	$0.1 \pm 0.3$
Red card	$0.0 \pm 0.1$	$0.0 \pm 0.1$

Table values contain the mean number of specific actions performed by a player during a single game section by gender.

### Supervised learning models

We included a representative subset of the main-stream models used in current literature for binary classification using numerical and categorical features and models with interpretable results. Logistic Regression models provide an extension of the linear regression model for classification problems and have been widely used in sports science. In addition, they offer probabilistic reasoning if correctly interpreted. To represent straight interpretable models, we added Decision Trees to the comparison. Decision trees capture the interaction between data features and provide natural dissemination of classes. Finally, due to the increasing availability and usability of machine learning libraries, Neural Networks are highly adopted by different applied research areas. However, Neural Networks are characterized by their uncertainty of behaviour, although performing at high accuracy levels due to their computing power. Thus, the inclusion of Neural Networks in this study aims to explore and showcase the explainability features of these high-performance computing algorithms. Furthermore, the results and interpretations could benefit from the overlapping of numerical reasoning and visual rules that the three algorithms provide.

Models were optimized using the Scikit-Learn machine learning library^28^ and PyCaret bundle^29^. Models performance was evaluated with Accuracy and AUC (Area under the Curve)^30^ metrics. AUC ranges between 0 and 1. A model whose predictions are 100% correct has an AUC of 1.0. Models optimization and training were performed in 70% of the total dataset while maintaining a 30% of the data set for evaluation. A randomized grid search^31^ was performed with a 10-fold stratified cross validation in the training dataset for model hyperparameters optimization. The same cross-validation design was used for each of the models training. The following sections describe each model’s algorithms and training and the selected techniques for explainability.

#### Logistic regression

We trained a Logistic Regression model using an L-BFGS solver with L2 penalty regularization and C=3.208, low tolerance at 0.0001, and 100 iterations. The model uses a logistic function to distill a linear equation’s output between two possible outcomes, 0 and 1. Therefore, feature weights cannot be interpreted as linear regressions because they do not influence the probability linearly. A strategy to analyze logistic regression feature weights relies on understanding the logistic regression model as a linear model for the log odds (probability of predicting class 1 divided by the probability of predicting class 0)^32^. A single change in the feature $x_i$ by one unit increases the log odds ratio by the feature’s corresponding weight, $\beta _i$. In other words, we can compute the prediction odds ratio for every feature by computing the corresponding weight exponent. The odds ratio indicates the effect of each feature in the estimated odds of the prediction.

#### Decision trees

The main advantage of Decision Trees against regression models for classification is to explore nonlinear relations between the label and the features and interactions between features. We designed the tree to split on each node by information gain and a minimum of 40 samples per node to retrieve meaningful sample of classified populations. We evaluated two interpretation methods for decision trees: Feature importance, measuring the impact of each feature on all the splits used, and the tree decomposition, visually assessing each path of the tree and the features involved. We used the *dtreeviz*^33^ python library to decompose and visualise the decision tree model. The overall feature importance in a decision tree can be computed in the following way: We measure the information gain on each split where the model uses the feature and aggregate the overall information gain of the tree-based model. All individual features are finally scaled to 100. The decomposition of a decision tree consists of starting from the root node, visit each of the next nodes while aggregating the rules extracted from each edge. The leaf nodes are the final prediction for the roles fulfilling every rule of the path.

#### Neural networks

Although deep learning has been very successful in optimization problems, it lacks openness and methods to understand how a classifier learns and make predictions. A single prediction potentially involves hundreds of operations. Therefore, current research is moving towards interpreting deep learning algorithms through model-agnostic interpretation methods. These methods do not depend on specific model classes and are applied after the model is trained. They usually analyze each feature’s impact on the output independently of the model’s structure. We implemented a basic neural network with 1 hidden layer, constant learning rate $\alpha =0.0001$, $\beta _1=0.9$ and $\beta _2=0.999$. We used the trained neural network to compute SHAP^34^ (SHapley Additive exPlanations) based on Shapley Values^35^. Shapley Values’s basic idea is to map the model’s prediction as a payout and the features as the game players. Shapley values then tell us how to distribute the payout among the features equitably. Computing Shapley Values requires a lot of computing power and time. That is why, in most real-world cases, only the estimated Shapley Value is feasible. SHAP estimates the contribution of each feature value to the prediction. We used a KernelSHAP explainer with 120 weighted k-medians of the original data set for the randomly sampled test predictions.

## Results

Models’ classification accuracy remained high through the three models implemented. In the following sections, we present each model evaluation and the main results extracted from the explainability methods implemented.

### Logistic regression

The Logistic Regression model yielded 95% of predicting accuracy and 0.99 AUC. The prediction odds have been defined as the probability of predicting class male (gender = 1) divided the probability of predicting class female (gender = 0). For instance, having an estimated odds of 4 means that the probability of the predicted label being male is four times as high as predicting females. Table 3 shows the top 10 features with their corresponding weights and odds ratio, ordered by odds ratio value. The odds ratio is the actual impact of each feature changes in the estimated odds. For instance, an increase in the number of ground duels increases the odds of male vs. female by a factor of 30.01. Table 3 shows that the most relevant features predicting class male more likely than class female prediction involve ground duels, shots, interceptions, and crosses.

In Table 4, we showcase the top 10 features with the estimated weights ordered by the absolute value of the weight. In this case, negative weights produce odds ratios lower than 1, indicating that the feature makes the prediction odds smaller. We can extract more distilled knowledge from the logistic regression models: Although ground duels increase the odds by a factor of 30, the volume of lost ground duels decreases the odds of male vs. female, indicating a higher relevance of lost ground duels for class female. The same reasoning can be applied to crosses’ values; it stands out that the number of crosses executed has a positive factor in the estimated odds, while unsuccessful crosses decrease the estimated odds.

**Table 3:** Logistic regression model top 10 features estimated weights and odds ratio ordered by odds ratio value.

Feature	Weight	Odds ratio
Ground duels	3.401654	30.013688
Shots off target	1.883792	6.578403
Shots on target	1.333649	3.794864
Interceptions	1.240091	3.455927
Successful crosses	1.142880	3.135788
Crosses	1.127932	3.089260
Free kicks	0.717951	2.050228
Goals	0.661713	1.938110
Aerial won	0.348462	1.416887
Successful passes	0.269733	1.309615

A single change in a feature by one unit increases the estimated odds (probability of predicting class male over the probability of predicting class 0) by a factor of its corresponding odds ratio.

**Table 4:** Logistic regression model top 10 features estimated weights and odds ratio ordered by the absolute weight value.

Feature	Weight	Odds ratio
Ground duels	3.401654	30.013688
Ground duels lost	− 2.935787	0.053089
Unsuccessful crosses	− 2.722266	0.065726
Clearances	− 2.267617	0.103559
Shots off target	1.883792	6.578403
Shots	− 1.663560	0.189463
Fouls received	− 1.565999	0.208879
Shots on target	1.333649	3.794864
Interceptions	1.240091	3.455927
Crosses	1.183030	3.264250

Negative weights produce odds ratio smaller than one, which indicates that predicting class male becomes less likely.

The training dataset for the model is composed of numerical and categorical data. For numerical features, odds ratios greater than one indicate that the prediction of class male vs. class female is more likely to occur. Odds ratios smaller than one show that the prediction of male vs. female is less likely to occur. The odds ratio interpretation slightly changes when analyzing categorical features; The odds ratio for categorical features compares the estimated odds when using each feature’s possible value. For each value of the variable, we have an odds ratio value that reflects the effect of that value among all the other possible values of the variable. As shown in the following tables, the logistic regression model also allows the analysis part to show each option’s effect in a categorical feature.

In Tables 5 and 6, we can observe that player position and game section impact the prediction’s likelihood. It is not easy to interpret the impact’s direction without considering the numerical variables of the dataset. However, we can state that Goalkeeper and Forward are the two positions that most change the prediction odds. In other words, goalkeepers and forwards contain more differences between genders than the other player positions available. Table 6 shows how the first and second half of the game impacts the estimated odds equally, but events done in overtime positively affect male vs. female predictions. We justify this effect since male games have more overtime periods than female games.

**Table 5:** Logistic regression model estimated weights and odds ratio for each possible value for Player Position.

Player position	Weight	Odds ratio
Goalkeeper	1.260011	3.525461
Substitute Midfielder	0.578782	1.783865
Defender	0.309324	1.362504
Substitute Goalkeeper	0.000000	1.000000
Substitute Defender	− 0.035701	0.964928
Substitute Forward	− 0.478682	0.619600
Midfielder	− 0.656056	0.518894
Forward	− 0.935120	0.392539

**Table 6:** Logistic regression model estimated weights and odds ratio for each possible value for Game section.

Game section	Weight	Odds ratio
Overtime-2nd Half	1.091627	2.979119
Overtime-1st Half	0.733228	2.081790
2nd Half	− 0.477029	0.620625
1st Half	− 0.516509	0.596600

### Decision trees

The Decision Tree model achieved 85% of accuracy while having a 0.9 AUC. We avoided overfitting by pruning the tree at nodes with a minimum of 40 nodes. Tree-based models are known for being straight forward interpretable and can yield coefficient-like mathematical evaluations of each feature. Table 7 shows the importance of the top 10 most relevant features in the final decision tree model implemented. The interpretation of each feature’s importance is different from the previous logistic regression model. If feature $x_j$ has an importance $w_j$, $x_j$ contributed $w_j$ to the model’s total information gain. In other words, the importance is an indicator of how much information the feature contains to disseminate between classes.

**Figure 2:**
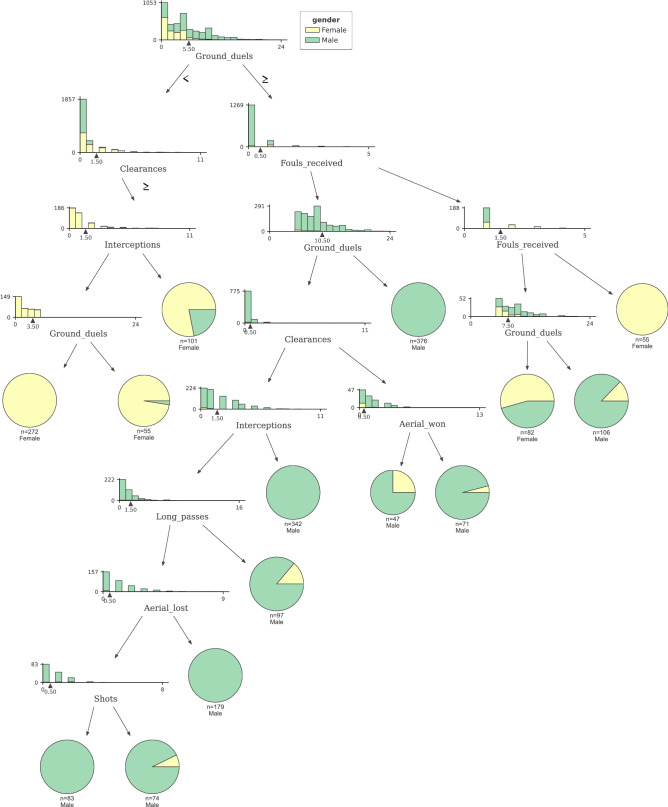
Visual representation of the pruned Decision Tree model. This figure shows the decision tree model’s root using ground duels as the highest entropy split. Early leaf nodes appear at the fourth level of the tree with high degree of purity.

Decomposing the decision path in a decision tree is an appropriate method to understand its predictions. The final decision tree model has a depth of 13 and contains 43 leaves. Figure 2 shows the surface level of the tree. The tree constructor selects the feature ground duels for the root split. At the third and fourth depth levels, we can already notice leaf nodes with a high purity level. The tree classifies 376 male class nodes correctly after ground duels value bigger than five, no fouls received, and ground duels bigger than ten. On the other side, 55 nodes with ground duels bigger than five and two or more fouls received are classified as female. Players with less than six ground duels, more than one clearance, and more than one interception are mostly classified as female.

**Table 7:** Feature coefficients representing the importance of each feature in the Decision Tree model.

Feature	Coefficient
Ground duels	0.308840
Fouls received	0.167490
Clearances	0.135580
Interceptions	0.118301
Unsuccessful passes	0.084388
Player Position: Goalkeeper	0.023302
Long passes lost	0.018937
Free kicks	0.015709
Successful passes	0.014780

The decision tree model assigns each feature importance depending on the information gain noted on the spit where the feature participates. The overall importance is scaled to 1.

### Neural networks

**Figure 3:**
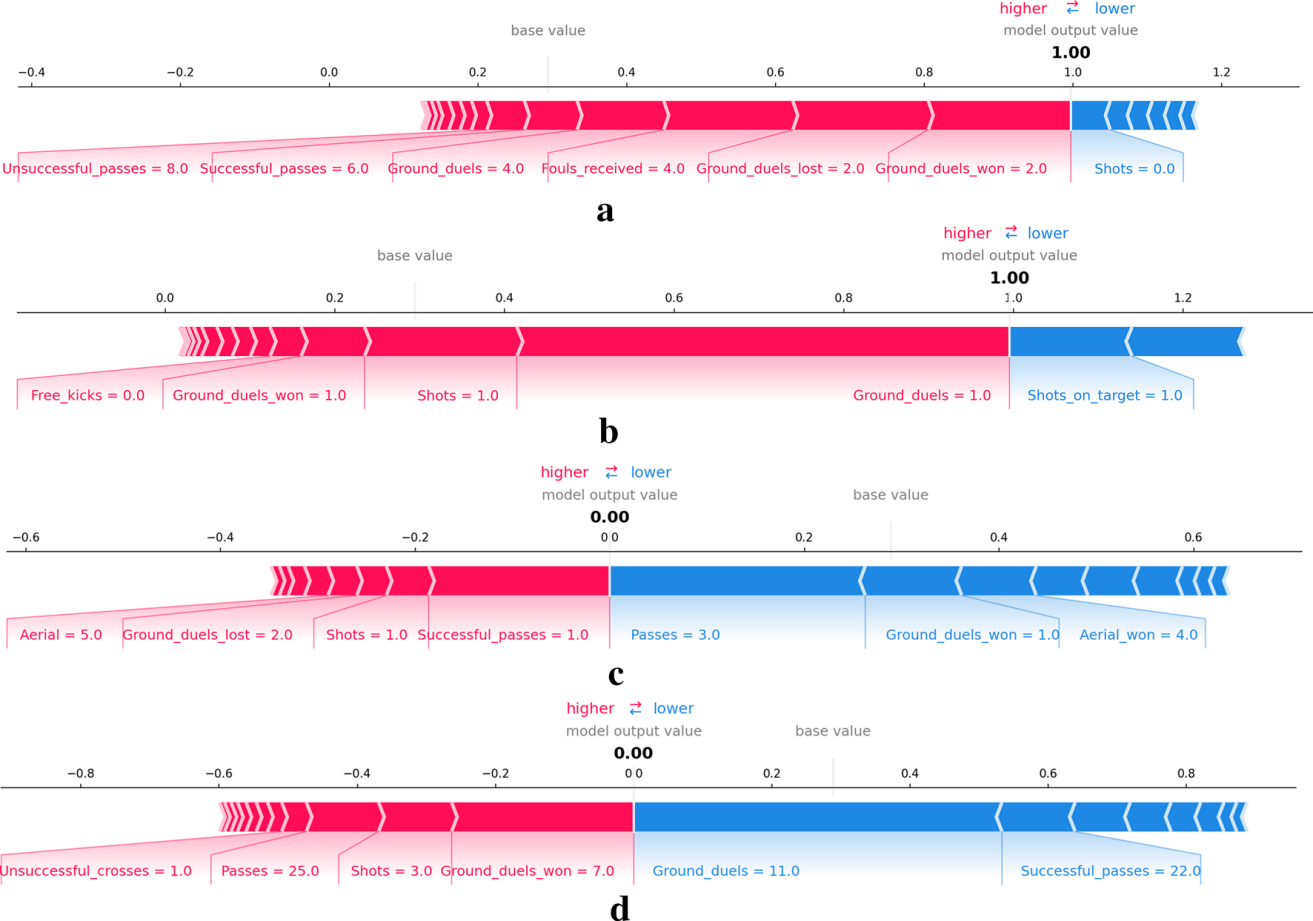
SHAP force plots explaining the predicted gender of four players of the data set with female class as the reference, the model output value is the probability of that instance to be classified as class female. The base value indicates the expected value for female classes. For female predictions (**a**) and (**b**), the explainer outputs a 1.00 probability while for male predictions (**c**) and (**c**) the probability is 0.00. Red arrows indicate positive Shapley values increasing the probability and blue arrows indicate negative values decreasing the probability.

The basic Neural Network model achieved the best results with 96% accuracy and AUC of 0.99. The KernelSHAP explainer interprets feature attributions to the prediction as forces that try to increase or decrease the prediction. In Fig. 3, we can see the individual decomposition of four instances in the data set into the sum of the effects of each feature value. The explainer describes the model taking the female class as the reference class and computing for each instance the probability to be classified as female. The base value is the expected output for the reference class or, in other words, the prediction if no information about the features was available.

Figure 3a,b are rows from the dataset that the model classifies as female. We can see how the model uses attributes such as clearances, fouls, ground duels, and passes to impact the output positively, and only ground duels impact the output in the opposite direction. On the other side, Fig. 3c,d show the effects leading to instances predicted as male.

**Figure 4:**
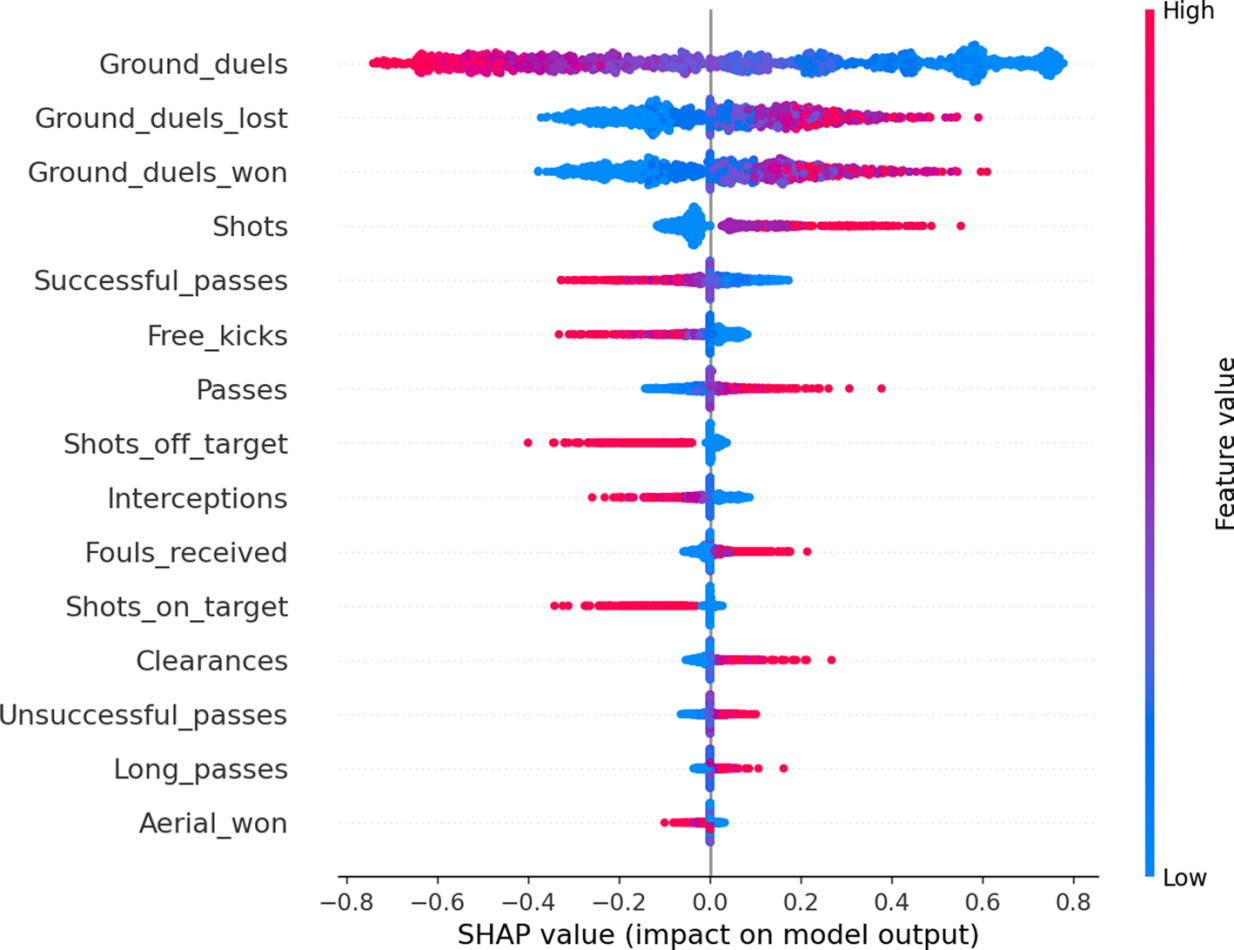
SHAP summary plot with the top 15 features ordered by feature importance. The class reference is defined as Female. A high number of ground duels increases the probability of the model classifying a female player. On the other side, high value of ground duels decreases the likelihood of prediction class Female, which is equivalent to increase the probability of predicting a male player.

Individual explanations might be useful to scratch the neural networks’ behavior, but they are too specific to particular instances of the data set. SHAP allows the aggregation of all the Shapley values to provide global explanations. We show the SHAP feature importance and direction of effects in Fig. 4. Ground duels remains as the most relevant feature as the other models also yielded. Moreover, a Female prediction is more likely with lower ground duels values. Interestingly, the Male class’s prediction is more probable as the number of successful passes increases while a higher number of total passes attempted increases the probability of predicting the instance as a female player.

**Figure 5:**
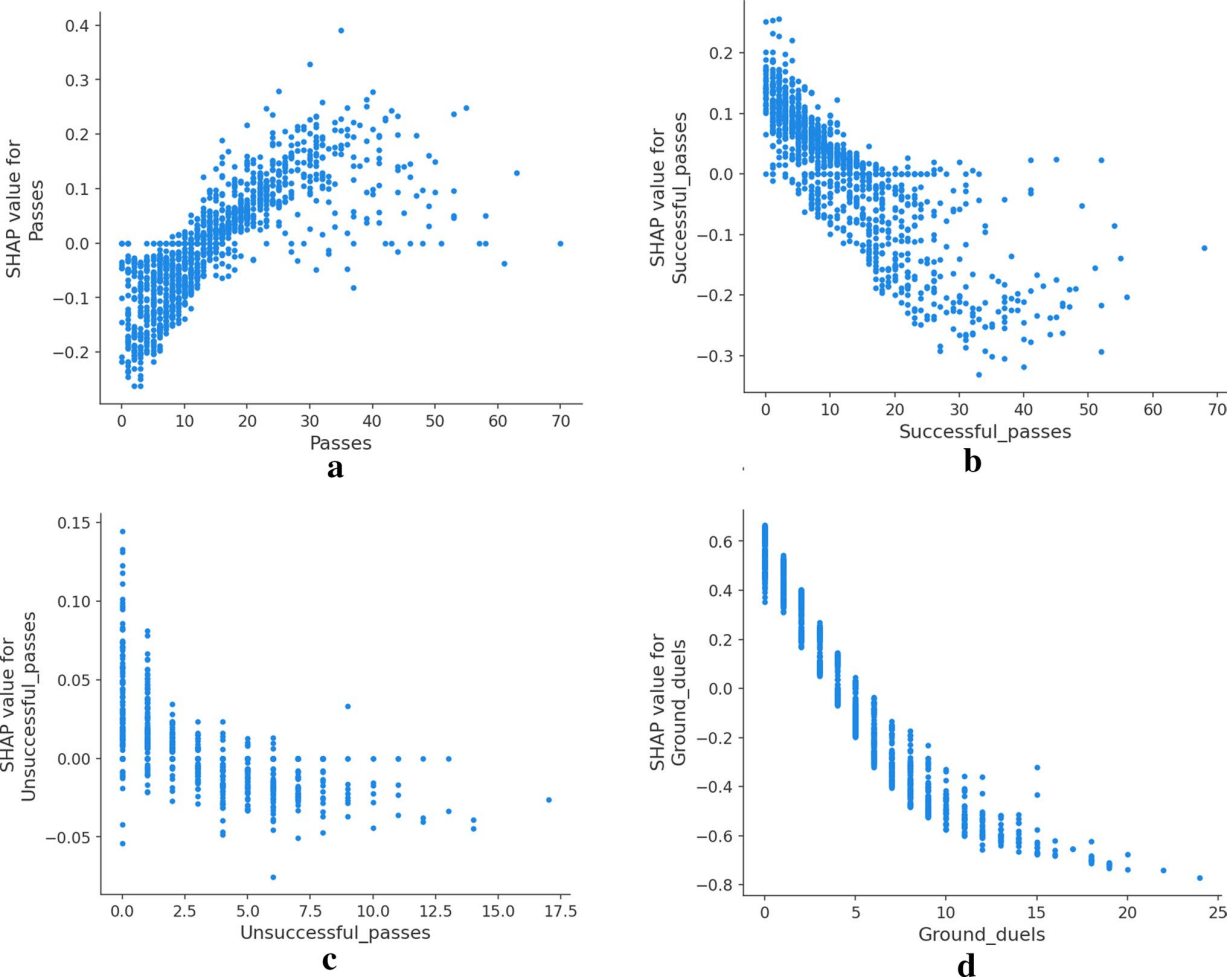

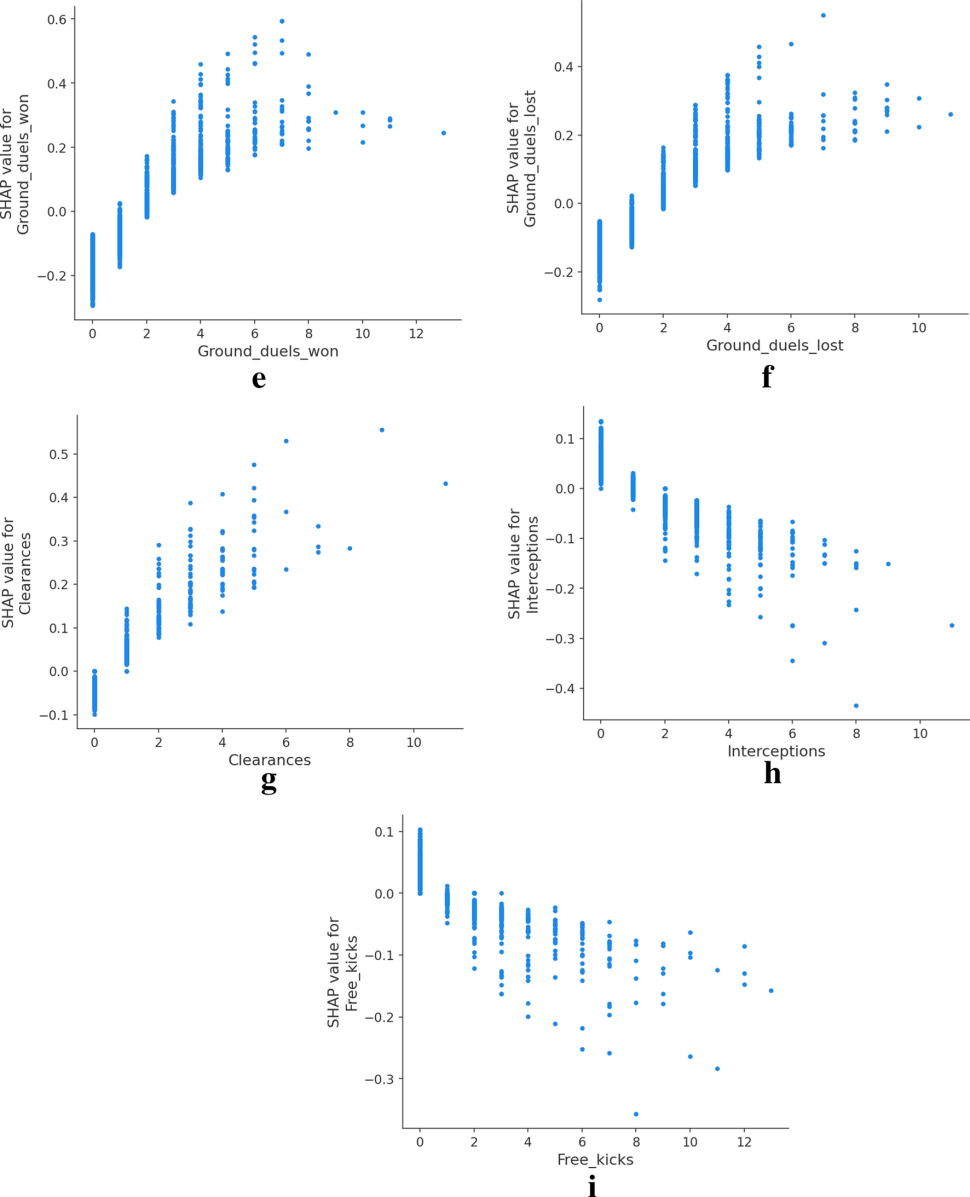
SHAP dependence plots showing the distribution of Shapley values as feature value growths. Using class Female as reference class. Higher SHAP values indicate higher probability of classification as Female.

The overall computation of Shapley values allows us to analyze how each feature values affect the output model. Several examples are presented in Fig. 5. It stands out how related features differ from each other when affecting the model classification. In the first row, Fig. 5a–c while a higher number of passes increases the probability of Female classification, better passes impact is inversed, denoting a possible trend of female players doing more but less accurate passes. On the other side, the number of ground duels decreases the probability of classifying as female, while the higher value in won ground duels makes them more likely to classify as male. A growing number of clearances further increases the probability of a female classification, while interceptions ot free kicks decrease the likelihood.

## Discussion

The aim of the present research was to identify differences between male and female players performance in European Football through machine learning supervised learning techniques. We proposed an end-to-end pipeline organizing two different football event data logs, data sets gathering all the actions during a European football game. The integrated dataset was computed by extracting each data source’s event-based metrics by game section and player position and harmonizing the overlapping features. We trained and evaluated three representatives supervised learning algorithms to classify the gender of each data point in the integrated dataset; Logistic regression, Decision Trees, and Neural Networks. The accurate classifiers were investigated by explainability methods to understand each model’s behavior. In the methodology presented, machine learning algorithms results are studied in detail, and the focus is moved to the underlying data and knowledge extraction rather than in the highly accurate predictions or outcomes. Several indicators have been highlighted as demonstrating differences between female and male performances, moreover, relationships between indicators and combination of actions in the game also have been shown to disseminate between genders. Independent conclusions and an integrated perspective with sports scientists would allow further discussion in possible similarities and differences and quality measurements in European football.

The overall predicting accuracy of the three models was satisfactory. Each model yielded good evaluation metrics and allowed the study to focus on the explainability methods. The Logistic regression produced feature weights to identify how the odds of male classification versus female classification were affected by each feature. We identified ground duels, shots, and interceptions positively impacting the odds of classifying males over females while higher values of lost ground duels, clearances, or shots decreased the odds. Moreover, we identify goalkeepers and forwards as the two most differing positions. As expected, the match section where actions were produced it is not significant to disseminate between male and female games. The decision tree findings presented the relations between match actions and identified patterns for classification. A fully functional tree of rules was developed, acknowledging groups of data points classified equally and following similar patterns. Data points were classified as male when ground duels value was bigger than five, no fouls received, and ground duels bigger than ten. Feature importance for each feature confirmed the same variables identified in the Logistic Regression results. Finally, we also explored model-independent methods to explain classification algorithms, SHAP methods allowed us to understand the classifications of a basic neural network. We analyzed the features with more effects in individual predictions, accumulative metrics, and the distribution of the feature’s impact in the model output depending on the feature value. Findings show that ground duels, fouls and passes where reafirmed to have an impact on determining the gender label. In addition, male perform more accurate passes and are involved in more ground duels. The three analysis concluded with common features involved as well as interesting combinations. Logistic regression and neural networks provide numerical effects for each feature, allowing us to rank and understand each feature’s relevance for gender classification. The addition of a rule-based tree reasoner complements the previous ranking with the interlinear elements and identifies patterns involving more than one feature.

The models understood the difference between male and female performance with high prediction accuracy without overfitting. However, this study is based on the assumption that no systematic difference exists in the data variables collected from the two data sources analyzed. Due to the scarcity of football data sources, collecting a sufficient amount of data is not trivial. Ideally, a single data source should incorporate male and female instances to thoroughly assess that the generation of the present event data logs from football games does not differ from male and female scenarios. Furthermore, it is essential to state that each model interpretability does not directly mean causality. The present study provides a data-driven analysis beyond traditional statistical analyzes and aims to extract trustful knowledge from machine learning algorithms. This study’s results could be applied to deploy similar analyzes with different pivotal attention, such as the player’s nationality, age, or other qualitative categories. Furthermore, domain expertise is needed to export the bits of knowledge presented in this study to profiled training programs.

## Data Availability

The original football event datasets used to obtain the data set during the current study are available from the corresponding author on reasonable request. Moreover, the dataset generated and analysed during the current study is available on figshare^36^.

## Code availability

The code for the preprocessing steps and data analysis pipeline performed for the study are available at https://git.io/JsmXK.
